# How getting noticed helps getting on: successful attention capture doubles children's cooperative play

**DOI:** 10.3389/fpsyg.2014.00418

**Published:** 2014-05-27

**Authors:** Nicola Yuill, Steve Hinske, Sophie E. Williams, Georgia Leith

**Affiliations:** ^1^Children and Technology Lab, School of Psychology, University of SussexBrighton, UK; ^2^Institute for Pervasive Computing, ETH ZurichZurich, Switzerland

**Keywords:** cooperation, play, audio, technology, joint attention

## Abstract

Cooperative social interaction is a complex skill that involves maintaining shared attention and continually negotiating a common frame of reference. Privileged in human evolution, cooperation provides support for the development of social-cognitive skills. We hypothesize that providing audio support for capturing playmates' attention will increase cooperative play in groups of young children. Attention capture was manipulated via an audio-augmented toy to boost children's attention bids. Study 1 (48 6- to 11-year-olds) showed that the augmented toy yielded significantly more cooperative play in triads compared to the same toy without augmentation. In Study 2 (33 7- to 9-year-olds) the augmented toy supported greater success of attention bids, which were associated with longer cooperative play, associated in turn with better group narratives. The results show how cooperation requires moment-by-moment coordination of attention and how we can manipulate environments to reveal and support mechanisms of social interaction. Our findings have implications for understanding the role of joint attention in the development of cooperative action and shared understanding.

## Introduction

Humans, it is claimed, evolved to cooperate to a greater extent than other primates, cooperating on a large scale and with a range of others, not just kin (Boyd and Richerson, 2009; Sterelny, 2012). In individual development, the ability to cooperate with others is both an end in itself and a driver of further social and cognitive development, according to the Vygotskyan Intelligence hypothesis (Moll and Tomasello, 2007). Deficits in elements of cooperative behavior, e.g., in referential pointing, the capacity for shared attention or language, may limit opportunities for social-cognitive development, for example in children with autism. An important context for developing the ability to cooperate is free play, and in particular, shared pretense, which requires joint planning, negotiation, problem-solving and goal-seeking (Bergen, 2002).

Recent research is illuminating about the age at which children acquire crucial components of cooperation, such as sharing attention and goals in toddlers, using short-duration, carefully-controlled experimental settings. We also know much about the broad consequences of cooperative play for social cognition in later childhood (e.g., Pellegrini, 1980; Lillard, 2001). However, we know little about how specific elements of behavior, such as attention sharing, might contribute to sustained cooperation in extended natural social interaction in childhood, beyond the point of initial skill acquisition. Children at this stage have acquired skills in attention, intention understanding and theory of mind, but how do they recruit and coordinate those skills in complex social interactions with peers? In the current studies, we investigate first the feasibility of manipulating the frequency of cooperative play using auditory stimuli (Study 1) and then the role of such stimuli in supporting cooperative play by manipulating the probability of successful attention-getting in triadic play sessions (Study 2).

In simple pared-down contexts, children can engage in joint attention over objects or ideas between 12–15 months (Bakeman and Adamson, 1984), use this skill in cooperating with shared goals from around 18 months of age (e.g., anticipating a heavily-laden adult's need for a door to be opened, Warneken and Tomasello, 2006), and toward the end of the second year, become able to work together with a peer to achieve a joint goal that cannot be reached alone (Brownell et al., 2006). In more naturalistic playful contexts, children show little cooperative play until 3 or 4 years later (Barbu et al., 2011). Early social play is dominated by solitary and parallel action: children pursuing similar activities alongside each other, (hence attending to others) but not integrating their actions, neither pursuing joint goals nor constructing shared pretense. If children show such competence in cooperation in toddlerhood, why is their play not characterized as cooperative until several years later?

Bakeman and Adamson (1984) point out the considerable gap that exists between infants' first display of joint attention over objects with mothers and their routine use of such coordinated attention in free play with peers. Thus we would expect a gap between the first mastery of shared attention and its recruitment into complex sequences of cooperative play, regardless of partner. Further, as Bakeman and Adamson point out, mothers in their study showed evidence of highly-structured scaffolding of their infant, for example, capturing attention by manipulating objects such as shaking a rattle, to make them “come alive” (ibid., p. 1281), compared to peers, who did not offer predictable or motivated support for joint attention. In this paper we argue that success in getting others' attention over objects is needed to support sustained cooperative play and that configuring objects to “come alive” by enabling play figures to utter context-relevant sounds can be used to manipulate successful attention capture and hence cooperative play.

The shift through development in frequency of parallel play to cooperative play relies on a change in the requirements of managing shared attention. Parallel play is defined by the one-sided monitoring of others' actions on objects without interacting, whereas cooperative play, which becomes predominant later in development, requires real-time management of mutual attention between partners over objects: one child observes another, as in parallel play, but also has to be involved, e.g., by responding to attention bids and capturing the other's attention, in order to engage in joint attention and action involving shared objects and ideas. If engaging others' attention is fundamental to engaging in cooperative play, then increasing the possibility of such engagement should increase the frequency of cooperative play.

Understanding and hence augmenting mechanisms of successful cooperation is a key aim of research in new technologies to support collaboration^1^ (Dillenbourg, 1999). One technology that can modify attention mechanisms is augmented reality, combining the physical and virtual to alter people's experiences of interaction. Tangible objects, such as toys, can be augmented with technology to support different forms of interaction. Such toys can increase children's motivation to play, partly through the engagement of novelty, but also through extending the ways children can interact through the toy (Farr et al., 2010). Such technology also provides a means of experimentally manipulating aspects of behavior, such as attention, in order to study the consequences for interaction. If these augmented toys do succeed in altering the potential for cooperative play through supporting joint attention, they provide an ideal opportunity to explore psychological questions: what mechanisms of attention are supported and how does attention capture relate to cooperation?

Auditory stimuli are particularly suited to coordinating attention in small groups. Audio provides a generally time-limited shared attentional focus that is hard to ignore, unlike visual cues that are constantly present, or touch cues that are specific to an individual rather than being shared experience. Furthermore, audio can be attended to in tandem with shared visual attention and shared action. The examples of attention-capture techniques reported in mothers of toddlers (Bakeman and Adamson, 1984) focus on audio: ringing a toy phone and shaking a rattle to attract and share attention. Research by Gogate et al. (Gogate et al., 2006; Matatyaho and Gogate, 2008) shows clearly that mothers of preverbal infants synchronously name and move an object, that mothers' actions are adapted to the linguistic capabilities of the infant, and that such actions can support word learning.

Children will be familiar with the use of audio-visual synchrony through their experience of early interactions with caregivers in early word-naming, and doubtless in more complex interactions with adults through early childhood, such as when adults use instructions while demonstrating complex tasks (e.g., teaching a child to tie a shoelace). It would therefore be no surprise if children were able to use such methods themselves to support the development of play narratives with peers. For example, constructing joint pretend play narratives with a set of figures could be supported by focusing playmates' attention on a specific figure in order to establish the figure as a main character and providing suggestive audio such as in-character speech or story suggestions in concert with moving the figure.

The Augmented Knights' Castle (AKC: Hinske et al., 2010) is an audio-enhanced Playmobil® medieval castle playset designed to let play figures “come alive.” The play figures are fitted with radio-frequency identification (RFID) tags that identify their location, such that placing a figure in a specific location produces a previously-programmed context-relevant sound: e.g., a ghost placed in the tower might howl, a knight might say “Shall we watch the jousting?” if placed in the courtyard or “Defend the castle!” if placed on the drawbridge. Hinske et al. argue from a design perspective that this multimedia content will support social play and engagement with the toy, and reinforces the arguments above about the role of audio in early interactions.

This paper presents two studies investigating how the AKC can be used to investigate the role of joint attention in supporting cooperative play in triads. Study 1 tests the basic proposal that audio enhancement will support cooperative play by comparing 6- to 11-year-olds playing in triads for a short period with the augmented toy compared with a non-augmented version. Study 2 assesses triadic play in augmented and non-augmented versions in more detail, to assess whether cooperative play is associated with more successful episodes of joint attention and to compare the quality of group products (narratives) arising from social play within the two different environments. This is a first step to understanding the potential role of getting noticed for cooperative play and the consequences of this for working with others.

## Study 1: comparing social play with augmented and non-augmented toys

### Methods

#### Participants and design

We analyzed video data of 48 6- to 11-year-old children (25 boys, 23 girls) in an archived dataset from the design evaluation stage (Hinske et al., 2010) collected in an elementary school of a small German town, with parental consent for video analysis. Children were randomly allocated to play in a triad^2^ in either the non-augmented (KC) or the augmented (AKC) version, with 8 groups of 3 (24 children) in each condition.

#### Materials

The playset was a Playmobil® Knights' Castle plus “Dragon Tower” set, and a small “enchanted forest” area, all mounted on a low rectangular base approximately 1 × 0.6m, with 30 figures and 9 locations (RFID readers) to pick up signals and generate sounds, giving the potential for over 200 different character-sound combinations, with sound effects (e.g., wolf howling) and speech (e.g., “I am the King”). The basic set comes with various moving parts, e.g., trapdoors, drawbridge.

#### Procedure

The children were videotaped in age-graded groups of 3 for 20 min of “playing with the toy as you would at home.” The augmented session was preceded by a very brief single demonstration that figures played sound when put in specific locations.

#### Coding and analysis

The play data was coded from video continuously using Mangold Interact software. The duration in seconds of each child's play behavior was coded using a slightly adapted version of the scheme used by Robinson et al. (2003), itself adapted from the classic scheme of Parten (1932). A second rater blind-coded 20% of the video, attaining an agreement Kappa of 0.75

Solitary play: This involves no orientation to other children, absorbed in the toy, with little or no eye contact with others and little or no apparent awareness of others' actions. The child appears focused on their own play. For example, a child facing away from peers, moving a play figure, with or without talking to the self.
Parallel/ other-oriented play: The child does not actively interact with playmates, observing them but not joining in. S/he may look at others but is not making direct eye contact. This also includes hovering watchfully near others. For example, a child might be holding a toy and observing the other two children engaging in play conversations or actions, but is not engaged with them.
Cooperative play: The child is involved in mutual engagement with a peer or peers, children may be passing and showing objects to each other, checking and responding to each other's actions. For example, two children may be holding a character each and having a play-fight, one child asks a question of another child, either in character or not.

There was a small number of occasions where children stepped out, e.g., to ask an adult for help, and these occasions were not included in the coding or total time measures.

This resulted in raw mean frequencies for the total time spent in each play type in each triad for the two playset conditions. Given that the session lengths were standard in length, the absolute duration figures were used in analysis. The average durations of each play type for triads of children in the two conditions were compared using analyses of variance (ANOVA) with SPSS statistical software.

### Results

**Figure 1:**
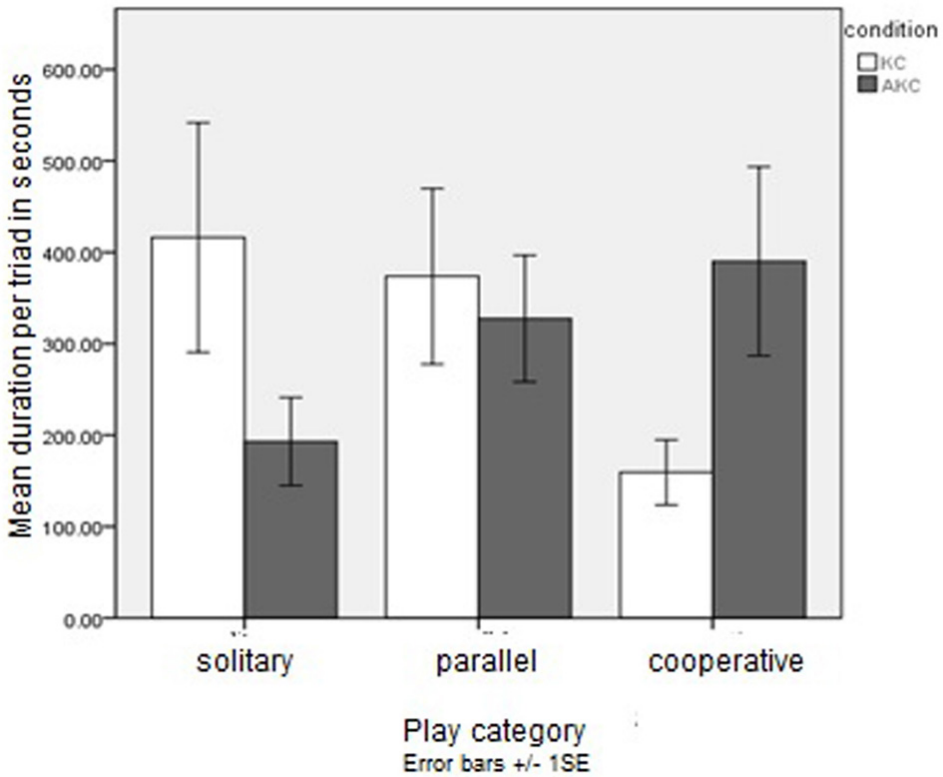
**Mean duration (SE) in seconds of different play types: non-augmented (KC) and augmented (AKC) toy: Study 1**.

Mixed ANOVA on durations of each play type across conditions yielded a significant Box's test, [*F*_(6, 1420)_ = 3.43, *p* < 0.01], indicating that the different categories might have different variances, violating assumptions for the chosen analysis. This was not improved by log transformation of the data, but given the equal sample sizes and lack of intercorrelation between the play variables this is not considered a serious problem (Howell, 2010). A MANOVA of play durations with toy type between subjects and the three play categories within subjects showed a significant interaction between play category and toy type, [*F*_(2, 28)_ = 10.50, *p* < 0.001, 05, η^2^ = 0.43], and a planned comparison supported the hypothesis that cooperative play would be more common for AKC than KC, [*F*_(1, 14)_ = 4.47, *p* < 0.05, η^2^ = 0.24], observed power of 0.51, as illustrated in Figure 1. Although solitary play duration was higher in KC than AKC, this difference was not significant, [*F*_(1, 14)_ = 2.76, *p* = 0.12], nor was there a significant difference between playset conditions for duration of parallel play, *F* < 1.

### Discussion

As predicted, children in the AKC condition played cooperatively for significantly longer than groups in the unaugmented condition: strikingly, more than double the length of time. Considering the brevity of the play session and the lack of a warm-up of the groups, who knew each other but were not chosen to be close friends, the absolute frequency of cooperative play with the AKC of around 40% of the session is notable. This difference does not appear to be the result of general motivation and engagement: the broader sample of children in the Hinske et al. (2010) study rated the toy and the play session highly overall (Hinske et al., 2010).

The results establish the initial claim that the augmented toy engenders more social forms of play and are consistent with the argument that the toy supports cooperation by more successfully capturing the attention of playmates. However, we did not directly measure whether attention bids were more successful in AKC sessions than KC ones. The play sessions were relatively brief, so it may be that children in the AKC condition were in a generally heightened state of attention because of novelty. Furthermore, we did not investigate the consequences of this increase in cooperative play for later interaction. In Study 2 we investigate how the outcome of bids for coordinated attention relate to the frequency of cooperative play in repeated and sustained play sessions, and how this would influence the quality of a group product, shared narrative, after the play sessions.

## Study 2. the role of attention capture in supporting cooperative play and joint narrative production

Study 1 established that the augmented toy produced more cooperative play than the non-augmented version in a short play session. In Study 2 we aimed to examine in more detail the potential precursors and consequences of such an increase in cooperative play. The first question is why such a clear increase occurred: more than twice the frequency of cooperative play in the augmented condition. We argued above that capturing attention through audio plays an important role in early mother-child interaction, and this is a capacity that is even more crucial when playing with peers, who may be less inclined to focus intently on scaffolding interactions with playmates than a supportive caregiver does with a young child. We maintained the challenge of play by having groups of three children rather than dyads, making for a higher level of competition for attention. If attention capture is the first step in supporting cooperative play, we should expect to see first that the audio augmentation is associated with increases in the success of bids for attention, and second, that greater success of attention bids predicts longer durations of cooperative play.

Cooperative play should also have consequences for group products of such play, such as a joint group story narrative, a task which we therefore added for this study. Garaigordobil (2006) cites Vygotsky's assertion that higher psychological functions emerge from social interaction, and more specifically, the idea that children's play leads to the development of imagination and creativity. Garaigordobil reports several intervention studies showing how a cooperative creative play program for 6- to 11-year-olds supports increased individual creativity post-intervention. We therefore predicted that if the AKC increased cooperative play, it should in turn support the production of group narratives judged to be more creative. We also investigated the textual qualities of the joint narratives. If the AKC encourages sharing and negotiating ideas, we would expect storytelling to show characteristics of mature storytelling (Cassell and Ryokai, 2001). In particular, Cassell and Ryokai argue that narrative construction in its more mature form consists of a mix of different types of utterance, including speaking in the role of characters, narrating events in the emerging story and negotiating about the progress of the narrative with one's co-creators. Joint story-telling requires complex moment-to-moment coordination of ideas to create a shared whole, even more taxing of coordination than cooperative play, and coherent narratives are associated with strong group cohesion (Eder, 1988). Greater frequency of cooperative play should therefore support more sharing of ideas, more mature narrative construction and hence more creativity in group story-telling.

To assess the potential precursors and consequences of cooperative play, we collected video data from triads of children playing with the KC or the AKC for two 30 min sessions over the course of a week, followed by the construction of a joint narrative. Video-recording from several angles enabled analysis not just of play type, as in Study 1, but also meant we could code each child's attempts to attract the attention of their peers to see whether successful attention-getting supported cooperative play. After playing, each group of three children was given time to construct a story which they then acted out for the experimenters, enabling us to investigate whether longer durations of cooperative play were associated with qualities of shared narratives. We therefore analyzed play type, as in Study 1, and added three further measures: the outcome, success or failure, of children's bids for playmates' attention, the range of narrative voices used in the joint storytelling and the creativity of joint narratives. The aim was to discover how small episodes of joint attention, prompted by the technology, might lead to the striking differences in play found in Study 1, and to assess what influence greater cooperative play would have on a shared product of interaction.

### Methods

#### Participants and design

Thirty-three children (20 boys, 13 girls, mean age 8 years 4 months, *SD* = 7.28 months) from a UK urban elementary school took part, comprising all children in two mixed Year 3–4 classes who provided parental and child consent. They played in mixed-gender groups of three, allocated by the teacher to fit in with children's work schedules. Six triads played with the AKC and five with the KC.

#### Materials

A modified playset was used, using the same Playmobil® materials, this time with castle, tower and forest on three separate irregularly-shaped “islands” with low bases, adding up to about the same area as the Study 1 set. There were around 20 characters, 9 of them with speaking roles at any of 12 positions, making 55 location-character combinations of sounds, including factual information, sound effects (dragon roaring), conversational turns (e.g., “Good day, Your Majesty”) and suggestions (e.g., “Defend the castle!”).

#### Procedure

The procedure was identical for the two conditions: the only manipulation was whether the Knights' Castle was played with in augmented form. Each group had two play sessions with the castle in the school library within a week. At the start of the first session, the group was told that they had a 30-min play session in which they could play “as they would at home.” For the AKC condition, sounds were briefly demonstrated. At the start of the second session, the group was told that they had another 20 min of free play, but that afterwards they were to plan a “short story” as a group, which would then be performed to the experimenters. Immediately after their free play, all groups were taken aside from the set and given photographs of the characters and locations to support planning and storyboarding. After 10 min the children were escorted back to the castle (now switched off in both conditions) and invited to perform their short story, with a sand-timer to mark when 5 min were up.

#### Coding and analysis

***Play states***. These were coded as in Study 1. An independent coder coded randomly-selected 20% of the sessions, attaining a Kappa of 0.87.

***Attention bids***. A section of 7 min of play, 5 min into the first session, was selected for detailed coding of attention bids. We judged that at this point in the session, children had settled into play but still needed to negotiate their patterns of interaction. For each child, we noted every occasion within the 7 min they made a bid to show or tell something to another child. Bids could be non-verbal (e.g., showing toy, seeking eye contact, smiling at peer while acting), or verbal (making sounds, suggestions). Each bid was coded as successful or failed, depending on whether either of the other children responded by looking, acknowledging or speaking. Inter-rater reliability on 10% of the data yielded a Kappa of 0.86.

***Narrative roles***. Cassell and Ryokai's (2001) scheme divides each utterance into one of three categories, with mature storytelling being characterized by a balance between them:

Narrative: telling the story, e.g., “There was a fight,” “The dragon attacks”
Character: speaking as one of the characters, e.g., “Good morning, Queen!”
Metanarrator: making a suggestion about the story, e.g., “Shall we make it a war?”

Inter-rater reliability computed on 100% of the sample gave a Kappa of 0.70.

***Creativity***. The creativity of the joint narrative produced by each group was coded using the scheme developed by Hennessey and Amabile (1988). Their creativity subscale requests ratings on 4 4-point scales (max = 16): creativity, liking, novelty and imagination. Our raters used a 3-point scale, giving a maximum score of 12, and two raters independently blind-scored each narrative, attaining a Kappa of 0.63.

### Results

#### Types of play

**Figure 2:**
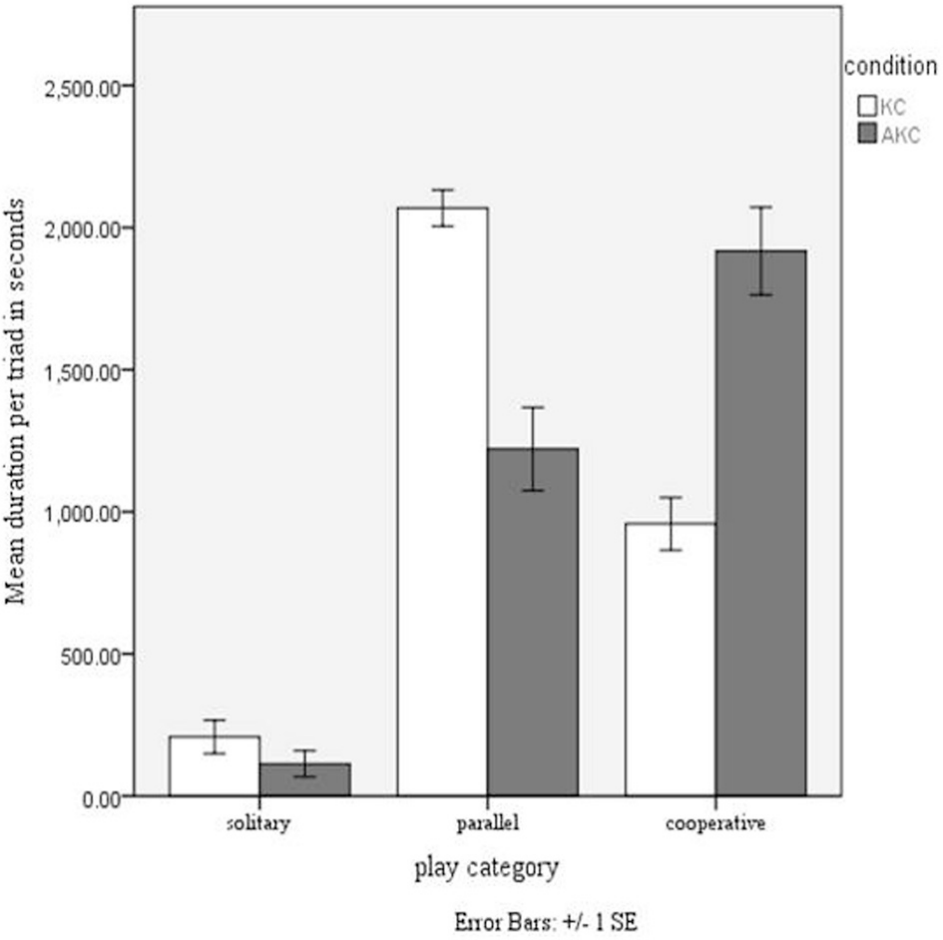
**Mean duration (SE) in seconds of different play types: non-augmented (KC) and augmented (AKC) toy: Study 2**.

**Figure 3:**
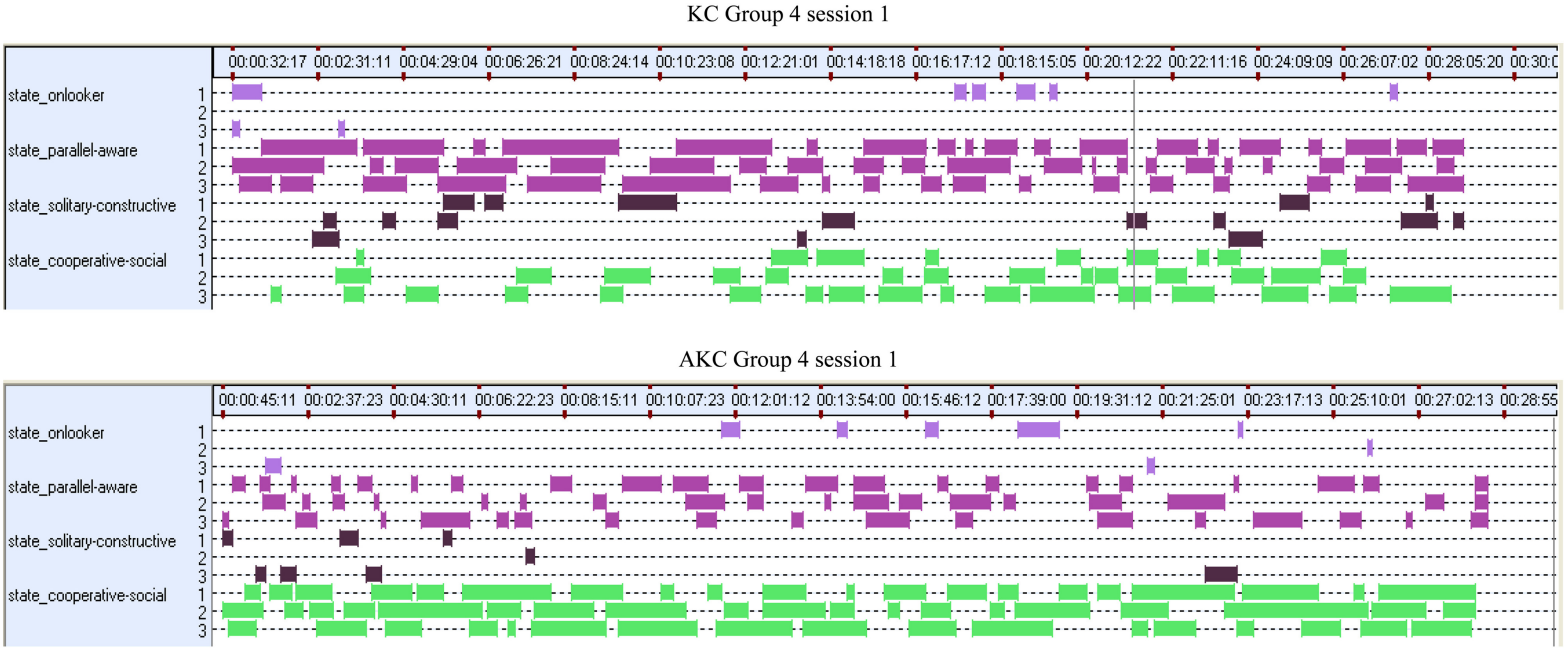
**Example INTERACT timelines for each child and each play type for KC (top) and AKC (bottom): the lowest three rows in green show patterns of cooperative play over time (x axis) for each child (y axis), with more frequent and longer bouts in AKC**.

We hypothesized that the increase in cooperative play with augmentation would be replicated with the different sample and extended play sessions of Study 2. We compared the mean durations in seconds for the three play behavior categories in each triad between the two playset conditions, using the total playing time of 55 min, as there were no striking differences in play pattern between the two sessions. As in Study 1, there was a significant interaction between playset and behavior, [*F*_(2, 18)_ = 33.82, *p* < 0.001, η^2^ = 0.79], observed power of 1.0 (see Figure 2). Using a stringent *p*-value of 0.001, we found cooperative play was more frequent in the AKC condition than in the KC, [*F*_(1, 32)_ = 71.48, *p* < 0.001]. In the KC condition parallel play was the most common play type, and significantly more so than in the AKC condition, [*F*_(1, 31)_ = 83.79, *p* < 0.001] There was no overlap between the two play conditions, when comparing confidence intervals for cooperative play, 95% CI for AKC [1628, 2205], 95% CI for KC [694, 1221], and for parallel play, 95% CI for AKC [970, 1471], 95% CI for KC [1840, 2297]. Solitary play was equally uncommon in both conditions. Typical timelines of play in the two conditions are shown in Figure 3, illustrating the longer duration of cooperative play, appearing as more frequent and more sustained bouts.

#### Attention bids

**Figure 4:**
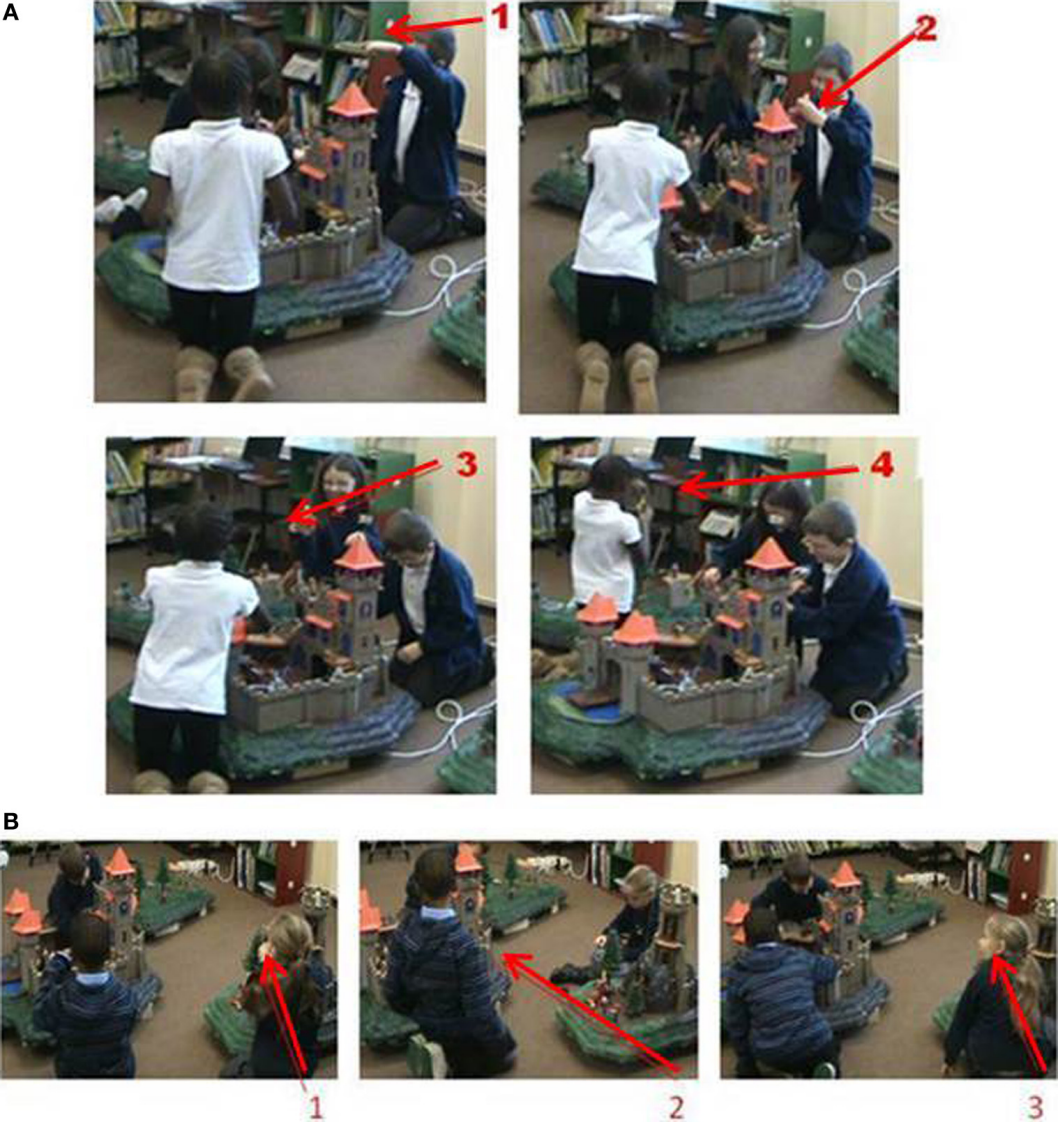
**(A)** Augmented toy. Boy (1) picks up and raises toy to tower to make sound, resulting in passing toy to girl (2) who then raises toy (3) and passes to girl (4), engaging all three. **(B)** Non-augmented toy. Girl (1) picks up toy to show boys, fails to capture attention (2), all three play separately despite girl's repeated attempt (3).

We assessed whether attention bids were more likely to succeed in the AKC than in the KC using a repeated-measures ANOVA with playset version between subjects and bid outcome (success or failure) within subjects (Box's M *p* > 0.05). Overall, bids were more likely to succeed than fail, and as hypothesized, the two playsets yielded different success rates: attention bids were significantly more likely to succeed in the AKC than in the KC condition, [*F*_(1, 28)_ = 7.99, *p* < 0.01, η^2^ = 0.22, power = 0.78]. In the AKC, there was a 68% chance of an attention bid being successful, compared to 52% with the KC. Sequences of stills from video (Figures 4A,B) illustrate a successful attention capture event in the AKC and an unsuccessful one in the KC.

If attention capture is important for sustaining cooperative play, and if the AKC serves as a specific booster of attention bids, then we would expect the success rate of attention bids to predict the amount of cooperative play with the AKC. This is indeed the case: the rate of successful bids (successful bids divided by total number of bids) was correlated with the frequency of cooperative play in the AKC, [*r*_(15)_ = 0.42, *p* < 0.06, and not correlated for the KC, *r*_(14)_ = −0.01].

#### Narrative role

**Figure 5:**
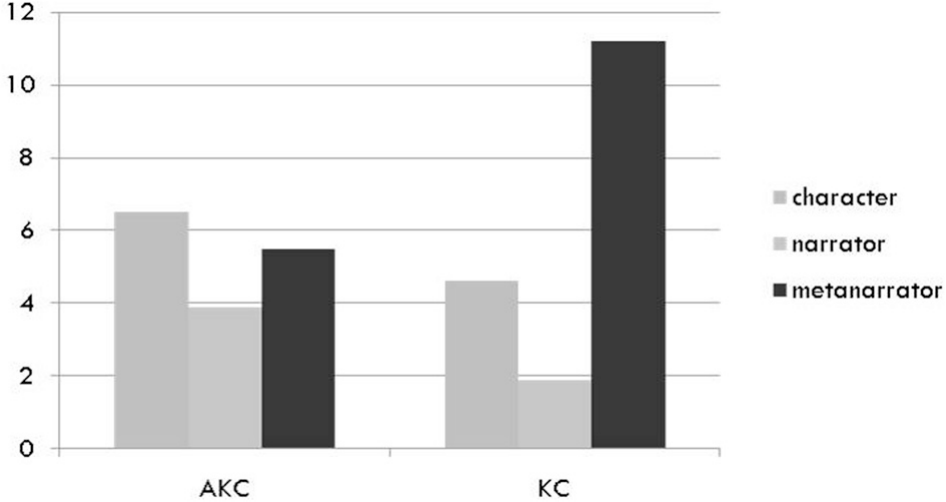
**Mean number of utterances of each play narration type in narratives with augmented (AKC) and non-augmented (KC) toy**.

We hypothesized that the AKC would support successful attention bids leading to cooperative play and that this type of play, in turn, should facilitate the construction of joint goals and shared pretense in negotiating storylines and joint planning, hence yielding more mature use of narrative roles. Figure 5 shows the distribution of different narrative roles: narrative story-telling, speaking in character and meta-narrations (making suggestions about the story).

A mixed ANOVA with playset between subjects and narrative role within subjects showed a significant effect of playset, [*F*_(3, 29)_ = 2.95, *p* < 0.05]. As shown in Figure 5, roles were more balanced in the AKC condition than in the KC, a pattern characterized by Cassell and Ryokai as “mature storytelling.” Children in the AKC made significantly more narrator statements, [*F*_(1, 31)_ = 4.68, *p* < 0.05, η^2^ = 0.13], and significantly fewer metanarrator ones, [*F*_(1, 31)_ = 4.2, *p* < 0.05, η^2^ = 0.12], than those in the KC. The domination of metanarrator talk in the KC condition reflects the fact that much time was spent negotiating, often unsuccessfully, what the play might be about, rather than actually narrating the story or talking in character.

The sample narratives below illustrate these differences. The AKC narrative includes all three narrative roles, including metanarrator (showing joint planning), narrator (which demands narrative coordination, if successful) and character, potentially supporting joint pretense. The KC example is typical in showing predominantly metanarrator comments—negotiation about characters and story themes, in this case fairly unsuccessful.

Sample narrative: Augmented castle (AKC) set

A: They left the king in complete ashes
B: (sings)
A: So they had to build a brand new castle, so they did
A: Once they had built the castle
B: Wait, wait
B: When the village heard that the crown was missing
A: The crowns gone missing everybody, me and my wolf will go over there and have a look, come on wolf
B: And [C], you need to be the king now, say, you say, what happened?
B: What happened to the castle, you say they built it new again
A: What happened to the castle?
C: They built it new again
A: You say, tell the story that happened 10 years ago, [C]
C: 10 years ago we had a big war and then they stole my crown
B: No, no, you don't say they stole my crown, you say I lost my crown under a tree somewhere
C: I lost my crown under a tree somewhere
A: Can we help you find it?
C: Yes
A: I will send my wolf to have a look around

Sample narrative: Non-augmented (KC) castle set

C: You two, I'm going to watch how you do it
C: If you two go on, I'm not gonna do it
A: Is he a baddie or goodie
C: That's not a baddie that's a goodie
B: That's a baddie
C: Goodie, goodie, goodie…
A: So they're there, and they wanna get there
A&B: (giggle)
A: Then all the baddies come
B: Then they all lived happily ever after
A: Ok so let's put the baddies in there, let's put the baddies back
B: He turns alive again, he turns alive again

#### Creativity

The KC group narratives received lower creativity scores (*M* = 7.25, *SD* = 1.40, maximum score of 12) than the AKC group (*M* = 9.20, *SD* = 1.68). Bearing in mind that the small sample size (6 vs. 5 groups) yields a statistical power of only 41%, a *t*-test shows [*t*_(9)_ = 2.10, *p* < 0.05], 1-tailed, with an effect size of Δ 1.12. Assigning the score of the relevant group play to each individual child, we observed a significant correlation between creativity of the play narrative and amount of cooperative play per child in the previous play sessions, [*r*_(33)_ = 0.72, *p* < 0.001], and a negative relation with parallel play, [*r*_(33)_ = −0.42, *p* < 0.05].

### Discussion

As in Study 1, the frequency of cooperative play was more than doubled with audio augmentation. The general pattern of play in Study 2 showed more parallel and less solitary play than in Study 1, and this is likely to reflect the longer and repeated play sessions used in Study 2. The additional analyses in Study 2 helped us to examine the reasons for the effect on cooperative play. Typical differences between patterns of play can be seen in Figure 3, illustrating the greater frequency and duration of bouts of cooperative play. We suggest that these more sustained sequences supported children in constructing relatively complex shared play sequences, with story themes emerging through the play session. This could explain why frequency of cooperative play in the play session predicted creativity of the joint narrative afterwards. Furthermore, viewing video sequences from a high camera angle suggested qualitative differences in physical movements between the two play sets: some groups in the AKC gave the impression of being tied together by invisible string, given the synchrony of their movements, whereas some KC sessions with low cooperation were characterized by children turned away from each other and moving asynchronously: further analysis of body posture and gaze could illuminate how movement coordination might support alignment of shared representations (Shockley et al., 2009).

We suggested that the audio augmentation would support social and pretend play by providing context-specific sound and speech: auditory information is ideal to support shared attention across individuals since it is shared simultaneously and supports shared visual attending to the sound source. It seems that adults facilitate young children's attention and word learning by naming and moving context-relevant objects, particularly for younger infants (Gogate et al., 2001). We speculate that this highly-scaffolded combination of context-relevant sound with object movements was effective in the current study to sustain a complex interconnected series of cooperative actions in a group of 3 peers, supporting the greater levels of cooperative play and play narratives. The AKC supported greater success in attention bids, and number of successful bids predicted frequency of cooperative play, in the AKC only. We argue that this is because the augmentation boosts attention and engagement, making the bid more likely to lead to further joint action, as in the illustration in Figure 4A. Getting the attention of two other peers is a tricky operation: shared audio is an effective way to do this, and the toys can be physically passed between the children to create coordinated sequences of play. Clearly, the current evidence shows inter-relations between attentional success, cooperative play and narrative quality, rather than direct causal mechanisms. The role of audio augmentation could be tested in within-subjects designs or by varying audio augmentation within-session to assess whether attention capture alters support for cooperative play dynamically. It would also be useful to attempt other means and modalities of varying attentional capture to assess whether the auditory modality is privileged over other means of attention-getting. We might also expect children's commitment to joint working to be shown in measures such as source monitoring for action, given young children's bias in overestimating their contribution to collaborative activity (Sommerville and Hammond, 2007).

### General discussion

The results of two studies in two different countries with age ranges from 6 to 11 years demonstrate that audio augmentation, providing shareable and salient audio attention-getters, produced more than double the rate of cooperative play than the same toy did when non-augmented. The technology allowed us to investigate the hypothesis that the contextually-variable audio would yield greater success in attention bids. We argued that getting noticed supports getting on together and Study 2 provided evidence for this: attention bids were more likely to succeed in the augmented condition than in the non-augmented and a higher rate of successful bids was associated with longer durations of cooperative play.

The cut and thrust of natural social interaction, whether play or work, requires at a minimum that group members share attention. We know that the capacity to share attention and joint goals emerges in toddlerhood, but cooperative play is uncommon until around 5 to 7. Cooperative play as studied here requires collaboration, “a coordinated, synchronous activity that is the result of a continued attempt to construct and maintain a shared conception of a problem” (Roschelle and Teasley, 1993, p. 95). This continued attempt to maintain shared understanding can only be achieved through recurring instances of sharing attention to and engagement with objects and ideas. The AKC provides a stimulus to joint attention because of the contextually-relevant audio. Our results suggest that as children become more skilled over development in learning how to gain the attention of others, their joint actions and plans can become better coordinated, and as we found, greater bouts of cooperative interaction are associated with more balanced and more creative narratives. Cross-species comparisons suggest that while some primates show elements of cooperation, they may not spontaneously use cues such as gaze direction for cooperation with humans (Warneken et al., 2006). This highlights the importance of investigating how abilities such as getting noticed are recruited in the service of cooperative endeavors. There will be within-child factors that support or detract from success of attentional capture and hence affect cooperative social interaction: for example, children with dyslexia have been reported to be relatively slow in automatic multimodal spatial attention (Facoetti et al., 2005). The complexity required to develop a shared understanding in a group emerges in the construction of joint narratives. Without the supportive augmentation, and hence with lower levels of cooperative play, children in the KC condition were generally unable to produce such coherent narratives, with a preponderance of non-consensual discussion about what the story might be, rather than managing to bring a story to fruition, and this was reflected in the creative quality of the play narratives, although the group difference in ratings was modest.

The results raise new questions about the nature and determinants of the development of cooperation through childhood. They also suggest a new method of “experimental technology” where different environments can be constructed to test out precise hypotheses about how different psychological mechanisms support interaction, and to produce new forms of behavior not otherwise possible, e.g., by revealing or concealing information about individual contributions to group efforts (Bachour et al., 2008; Kreitmayer et al., 2012). We know that environmental factors influence development of cooperation over time (Howes and Matheson, 1992) but the present study identifies some types of psychological mechanisms through which environmental factors must have their effects. For example, “poor” childcare settings might involve more distractions that work against successful attention-getting in extended group interactions, while “good” settings might support attentional strategies through a judicious mix of novel toys. On the other hand, for groups who have attentional difficulties and in particular atypical patterns of attention to other people, as in autism (Mundy et al., 1990), an analysis of environmental support for shared attention and hence cooperation might require less novelty and more time for habituation to an environment. Understanding more about such mechanisms of interaction has clear practical implications for designing environments to support interaction (Yuill and Rogers, 2012).

Our findings concern not just “child's play,” important though that is for development and learning (Hirsh-Pasek and Golinkoff, 2008). They also need to be considered in relation to collaborative behavior in work settings and with adults. The macroscopic behavior of cooperation is underpinned by micro-moments of successful attention bids. The more attention bids succeed, the more likely is cooperation. Recognizing this “snowball” effect should help us understand more about how joint attention skills are recruited into longer sequences of interaction and about the consequences of cooperative interaction for group productivity. Capturing attention over shared objects and ideas seems to provide social glue that binds together interactants, at which point more complex verbal negotiation can be involved in building shared understanding, and other mechanisms, such as linguistic alignment (Fusaroli et al., 2012) come into play.

The results also suggest ideas for interventions to support collaboration. Given that neither every location nor every figure in the AKC was tagged for sound, there were plentiful instances of children in the AKC condition capturing a peer's attention when a sound was not produced: for example, holding a toy high above the set meaning the tag was not detected and hence no sound was triggered. In these cases children sometimes used visual cues (e.g., waving the figure) and sometimes used audio (e.g., making a sound effect themselves or speaking to others to direct attention). Children did not appear to use the AKC in a deterministic way of seeking sound-action correspondences: such correspondences occurred in a more happenstance way, meaning that the sounds functioned as a trigger to engage children in shared audio experiences, creating an environment with an overall high level of attentiveness to other children's actions. This situation, in which there was simply the potential for context-specific effects, could make play intrinsically more engaging and attention-capturing for children. Future work should examine factors such as timing, number, acoustic qualities, and content of auditory cues in supporting attention in peer groups. Play benefits might be achieved by other more static novel features of a toy, but the advantage of the AKC is that the range of effects maintains novelty, especially as the set can be configured in any way the user desires within certain broad parameters and can alter dynamically over time, e.g., changing the probability of specific sounds. Harnessing attention might be used productively to support collaboration in many different settings.

## Conflict of interest statement

The authors declare that the research was conducted in the absence of any commercial or financial relationships that could be construed as a potential conflict of interest.

## Footnotes

^1^Technology literature makes a distinction between cooperation and collaboration. Here “cooperation” is used, to match terminology in developmental psychology: within this paper terms are used synonymously.

^2^The original data comprised dyads and triads: triads provide a greater attentional and cooperative challenge than dyads, where it is easier to get a partner's attention, so we analyzed only triads.
